# Undernourished children presenting to an urban emergency department of a tertiary hospital in Tanzania: a prospective descriptive study

**DOI:** 10.1186/s12887-019-1706-1

**Published:** 2019-09-11

**Authors:** Prosper J. Bashaka, Hendry R. Sawe, Victor Mwafongo, Juma A. Mfinanga, Michael S. Runyon, Brittany L. Murray

**Affiliations:** 1 Emergency Medicine Department, Muhimbili University of Health and Allied Sciences, P.O Box 65001, Dar es Salaam, Dar es salaam Tanzania; 2 Emergency Medicine Department, Muhimbili National Hospital, Dar es Salaam, Tanzania; 3 Department of Emergency Medicine, Carolinas Medical Center, Charlotte, North Carolina USA; 4 Division of Paediatric Emergency Medicine, Emory University School of Medicine, Atlanta, GA USA

**Keywords:** Emergency department, Malnutrition, Paediatrics, Sub-Saharan Africa

## Abstract

**Abstract:**

Background: Childhood undernutrition causes significant morbidity and mortality in low- and middle-income countries (LMICs). In Tanzania, the in-hospital prevalence of undernutrition in children under five years of age is approximated to be 30% with a case fatality rate of 8.8%. In Tanzania, the burden of undernourished children under five years of age presenting to emergency departments (EDs) and their outcomes are unknown. This study describes the clinical profiles and outcomes of this population presenting to the emergency department of Muhimbili National Hospital (ED-MNH), a large, urban hospital in Dar es Salaam, Tanzania.

**Methods:**

This was a prospective descriptive study of children aged 1–59 months presenting to the ED-MNH over eight weeks in July and August 2016. Enrolment occurred through consecutive sampling. Children less than minus one standard deviation below World Health Organization mean values for Weight for Height/Length, Height for Age, or Weight for Age were recruited. Structured questionnaires were used to document primary outcomes of patient demographics and clinical presentations, and secondary outcomes of 24-h and 30-day mortality. Data was summarised using descriptive statistics and relative risks (RR).

**Results:**

A total of 449 children were screened, of whom 34.1% (*n* = 153) met criteria for undernutrition and 95.4% (*n* = 146) of those children were enrolled. The majority of these children, 56.2% (*n* = 82), were male and the median age was 19 months (IQR 10–31 months). They presented most frequently with fever 24.7% (*n* = 36) and cough 24.0% (*n* = 35). Only 6.7% (*n* = 9) were diagnosed with acute undernutrition by ED-MNH physicians. Mortality at 24 h and 30 days were 2.9% (*n* = 4) and 12.3% (*n* = 18) respectively. A decreased level of consciousness with Glasgow Coma Scale below fifteen on arrival to the ED and tachycardia from initial vital signs were found to be associated with a statistically significant increased risk of death in undernourished children, with mortality rates of 16.1% (*n* = 23), and 24.6% (*n* = 35), respectively.

**Conclusions:**

In an urban ED of a tertiary referral hospital in Tanzania, undernutrition remains under-recognized and is associated with a high rate of in-hospital mortality.

## Background

Childhood undernutrition is a common and serious health problem affecting children under the age of 5 years [1, 2]. Undernutrition is a devastating condition which affects the entire body; this predisposes children to opportunistic infections and severe illness. Acute undernutrition is known to be complicated by life-threatening medical conditions such as dehydration and shock, hypoglycaemia, hypothermia, electrolyte abnormalities, and micronutrient deficiencies [3–5].

In low- and middle-income countries (LMICs), children have high rates of undernutrition, and there are several identified risk factors for undernutrition: unemployment, living in a rural area, poor hygiene, low levels of maternal education, long distances to health care, poor socio-economic status, a large number of siblings in the family, cessation of breastfeeding before six months, poor immunization history, unrepaired or undiagnosed congenital disease, and disease conditions such as HIV/AIDS [6, 7–9]. Most healthcare facilities in LMICs use the World Health Organization (WHO) recommended 10 step protocol [3] in the management of undernourished children [1, 4, 10, 11]. This protocol calls for prompt management of dehydration and/or shock, hypoglycaemia, and hypothermia, the treatment of infection, the correction of micronutrient deficiencies and electrolyte abnormalities, and the careful reintroduction of feeding, along with rehabilitation, emotional support, time for recovery, and a follow-up plan [3]. This WHO protocol has been shown to improve mortality rates of undernourished children under five years of age in LMICs [1, 11]. However, these guidelines focus on longitudinal hospital management, and the initial management of acutely ill undernourished children in EDs in LMICs remains variable in terms of care, resources, and local protocols [4, 11, 12].

In Tanzania, emergency medicine is a relatively new specialty and Muhimbili National Hospital (MNH) is the only public hospital with a full-capacity ED. In the ED-MNH, physicians have the resources for early recognition, stabilisation, treatment, and proper disposition of acutely ill undernourished children [13–15].

Prior to this study, the burden and outcomes of acutely ill undernourished children at the ED-MNH were unknown. We aimed to describe the clinical profiles and outcomes of these acutely ill, undernourished children under five years of age who presented to the ED-MNH. Our results provide an opportunity to begin to define the role of emergency medicine providers in the initial recognition and management of acutely ill, undernourished children.

## Methods

### Study design

This was a prospective descriptive study conducted from July 4, 2016 to August 31, 2016. Undernourished children aged 1–59 months presenting to the ED-MNH were included in our study after obtaining parental consent.

### Study setting

MNH is located in the Ilala municipality in the city of Dar es Salaam and serves as the national tertiary referral hospital in Tanzania. It is the only public hospital with a full-capacity ED in the country. The ED-MNH opened in 2010 and is currently the only training site in Tanzania for the degree of Master of Medicine in Emergency Medicine. The ED-MNH sees 150 to 200 acutely ill patients (adults and children) each day, one fourth of whom are children under 18 years of age. There are dedicated paediatric treatment and resuscitation rooms where nurses, general practitioners, and resident physicians provide care that is overseen by emergency medicine physician specialists.

### Sample size calculation

We calculated that a sample size of 135 subjects would allow us to characterize the mortality rate among our study population with a 95% confidence interval (CI). Our assumptions for this calculation were extrapolated from published data from Mwanza, Tanzania by *Ngallaba* et al. [1] that showed a case fatality rate of 8.8% amongst undernourished children. We further adjusted the sample size to account for up to a 10% loss to follow-up.

### Study procedure

A consecutive sampling technique was used for enrolment. Paediatric patients aged between 4 weeks and 59 months presenting to the ED-MNH during pre-selected research shifts for 8 weeks were screened for undernutrition using the WHO Z-score criteria for undernutrition, which are Weight for Height/Length (WFH/WFL), Height for Age (HFA), or Weight for Age (WFA) less than one standard deviation (< −1SD) below established mean values. The most significant Z-score for each patient was used to classify the severity of their undernutrition [6]. The principal investigator or a trained research assistant initially obtained verbal consent from each parent/caregiver to screen children for eligibility. For those who were eligible, written consent was obtained. Patient demographics (age in months, sex, residences, and referral status), clinical presentation, physical exam findings, initial interventions, and outcomes were documented both from the caretaker interviews and the electronic medical record for all enrolled patients. The children’s body weights were measured using a standardised digital scale, heights were measured using a measurement height/length board in conscious children above two years, and length was measured in children less than two years and all unconscious children using a length board. Z-scores for WFH/WFL, HFA, and WFA were calculated and compared to the WHO standardised growth chart to interpret the severity of undernutrition. For each patient, their most significant Z-score was used to classify their undernutrition. They were classified as severe for a Z-score < − 3 SD, moderate for a Z-score between − 3 SD and < − 2 SD, and mild for a Z-score between − 2 SD and < − 1 SD. ED final diagnoses were recorded for all children enrolled in the study and all children were followed up through their hospital stay by evaluation of their medical records, and through direct cell phone calls to their caregivers at 24 h, 48 h, and 30 days after their ED visits to determine their outcome. ED providers were blinded to the study investigator’s findings.

### Study outcomes

The primary outcomes included identifying the proportion of acutely ill children under five years of age presenting to the ED-MNH that were undernourished, establishing whether or not undernutrition was being documented as an ED physician diagnosis, and determining the in-ED mortality of undernourished children. Secondary outcomes included determining the 24-h, 48-h, 30-day, and overall hospital mortality of acutely ill children with undernutrition.

### Data analysis

Data were analysed with StatsDirect version 3.0.133 (Stats Direct Ltd., Cheshire, UK). Descriptive statistics (counts, median, interquartile ranges and percentages) were generated for demographic characteristics, clinical features, and outcomes of patients in our study. Inter-group comparisons were made using the Chi Square or Fisher’s exact tests, relative risks, and 95% confidence intervals (CIs) [16]. Two-sided *p*-values < 0.05 were considered significant.

## Results

### Demographic characteristics and classification of undernourished children under five years of age

**Fig. 1:**
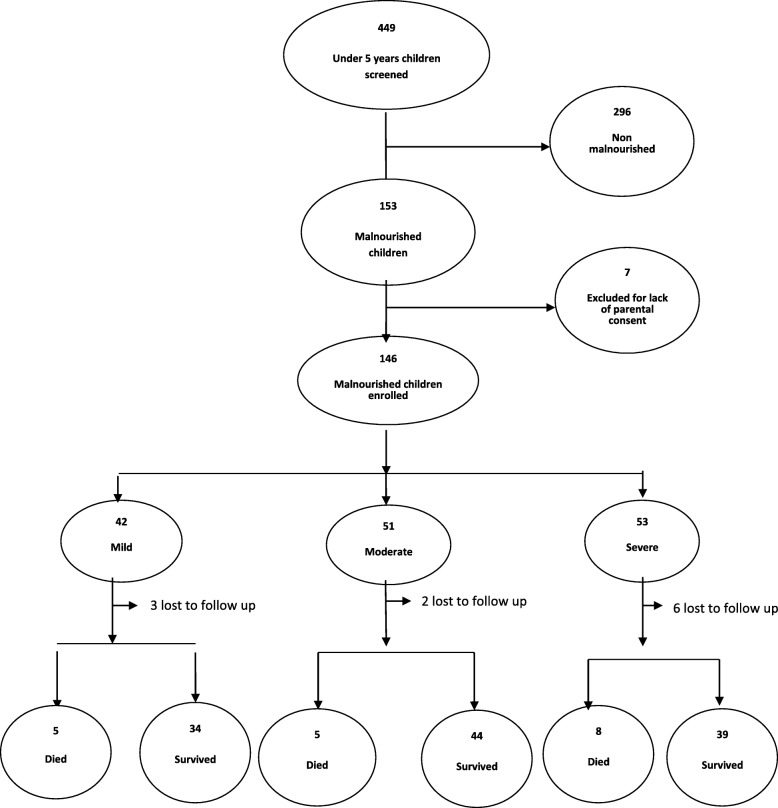
The study flow chart showing the enrolment of children under five years of age, malnutrition classification, and their outcomes

A total of 449 children under five years of age were screened throughout the eight-week study period; 34.1% (*n* = 153) were identified as undernourished. Among those children, parents of 95.4% (*n* = 146) consented to be in the study and were enrolled (Fig. 1). Among those enrolled, 28.8% (*n* = 42) had mild, 34.9% (*n* = 51) had moderate, and 36.3% (*n* = 53) had severe undernutrition**.**

The majority of children were male 56.2% (*n* = 85) with a median age of 19 months (IQR = 10–31 months). Almost one third, 31.5% (*n* = 46), were between one and twelve months of age. Most caretakers were biological mothers (90.4%, *n* = 132) and had at least primary school education (89%, *n* = 130). Low socioeconomic status (as evidenced by self-identification as peasants and/or housewives) was identified in 66.5% (*n* = 97) of caretakers. Most patients, 68.5% (*n* = 99), were referred from peripheral health facilities and 44.5% (*n* = 65) were ill for more than two weeks before presenting to the ED-MNH. The majority of patients, 65.1% (*n* = 95), resided in the city of Dar es Salaam (Table 1).

**Table 1:** Demographic characteristics and classification of undernourished children^a^

Characteristics	Overall (*N* = 146) n (%)	Mild (*N* = 42) n (%)	Moderate(*N* = 51) n (%)	Severe (*N* = 53) n (%)
Age in groups				
1–12 month	46 (31.5)	8 (19.0)	13 (25.5)	25 (47.2)
13–24 month	39 (26.7)	16 (38.1)	16 (31.4)	7 (13.2)
25–36 month	29 (19.9)	6 (14.3)	13 (25.5)	10 (18.9)
37–48 month	20 (13.7)	5 (11.9)	7 (13.7)	8 (15.1)
49–59 month	12 (8.2)	7 (16.7)	2 (3.9)	3 (5.7)
Child’s sex				
Male	82 (56.2)	22 (52.4)	31 (60.8)	29 (54.7)
Duration of illness				
More than 2 weeks	65 (44.5)	18 (42.9)	16 (31.4)	31 (58.5)
Referred status				
Referred	100 (68.5)	31 (73.8)	26 (51.0)	43 (81.1)
Self referral	46 (31.5)	11 (26.2)	25 (49.0)	10 (18.9)
Residence				
Dar es Salaam	95 (65.1)	29 (69.0)	36 (70.6)	30 (56.6)
Other	51 (34.9)	13 (31.0)	15 (29.4)	23 (43.4)

^a^Z-scores for WFH/WFL, HFA, and WFA were calculated and for each patient. The most severe Z-score for each child was used and interpreted as severe for a Z-score < −3 SD, moderate for a Z-score between − 3 SD and < − 2 SD, and mild for a Z-score between − 2 SD and < − 1 SD

### Clinical characteristics associated with undernutrition in children under five years of age

Fever was the most common chief complaint among patients in our study and was present in 24.7% (*n* = 36) of cases. On examination, 19.9% (*n* = 29) of the children had fever (> 38.3 °C), 24.6% (*n* = 35) had tachycardia for their respective age, and 16.1% (*n* = 23) had a decreased level of consciousness (GCS < 15). Among children who had laboratory tests performed, hyponatremia (sodium < 132 mmol/L for children below 12 months, < 136 mmol/L for children 12 months and above) was found in 74.4% (n/*N* = 29/39) and anaemia (haemoglobin < 10.9 g/dl) was detected in 77.1% (n/*N* = 37/48) of patients. Fifty-eight children (39.7%) had pre-existing chronic disease, 29 had congenital heart disease and 19 had cerebral palsy (Table 2).

**Table 2:** Clinical characteristics with classification of undernutrition^a^ in children

Abnormal clinical findings	Overall (*N*^b^ = 146) n/N (%)	Mild (*N* = 42) n/N (%)	Moderate (*N* = 51) n/N (%)	Severe(*N* = 53) n/N (%)
Chief complaints				
Fever	36/146 (24.7)	14/42 (33.3)	13/51 (25.5)	9/53 (17.0)
Coughing	35/146 (24.0)	9/42 (21.4)	12/51 (23.5)	14/53 (26.4)
Difficulty in breathing	26/146 (17.8)	7/42 (16.7)	7/51 (13.7)	13/53 (24.5)
Failure to thrive	15/146 (10.3)	2/42 (4.8)	7/51 (13.7)	6/53 (11.3)
Lower limb swelling	9/146 (6.2)	2/42 (4.8)	4/51 (7.8)	3/53 (5.7)
Vital signs				
Hypoxia (SPO_2_ < 90%)	9/144 (6.3)	1/41 (2.4)	2/50 (4.0)	6/53 (11.3)
Bradypnea	8/143 (5.6)	5/41 (12.2)	2/50 (4.0)	1/52 (1.9)
Tachycardia	35/142 (24.6)	11/41 (26.8)	14/49 (28.6)	10/52 (19.2)
Temperature > 37.5 °C	29/146 (19.9)	8/42 (19.0)	9/51 (17.6)	12/53 (22.6)
Low AVPU score (GCS < 15)	23/143 (16.1)	8/42 (19.0)	8/48 (16.7)	7/51 (13.7)
Point of care tests (POCs)				
Hypoglycaemia < 3.5 mmol/dl	3/58 (5.2)	1/16 (6.3)	1/15 (6.7)	1/27 (3.7)
Hypokalaemia	12/39 (30.8)	2/11 (18.2)	5/12 (41.7)	5/15 (33.3)
Hyponatremia	29/39 (74.4)	8/11 (72.7)	9/12 (75.0)	12/16 (75.0)
Haemoglobin (< 10.9 g/dl)	37/48 (77.1)	13/15 (86.7)	11/13 (84.6)	13/20 (65.0)
Child’s chronic disease				
Congenital heart diseases	29/146 (19.9%)	4/42 (9.5%)	8/5 1 (15.7%)	17/53 (32.1%)
Cerebral palsy	19/146 (13.0%)	4/42 (9.5%)	7/51 (13.7%)	8/53 (15.1%)
Hydrocephalus	5/146 (3.4%)	0	4/51 (7.8%)	1/53 (1.9%)

^a^Z-scores for WFH/WFL, HFA, and WFA were calculated and for each patient. The most severe Z-score for each child was used and interpreted as severe for a Z-score < −3 SD, moderate for a Z-score between −3 SD and < −2 SD, and mild for a Z-score between −2 SD and < −1 SD

^b^**N** represents the total number of children in each column that had the test/variable recorded

### Disposition and mortality of undernourished children under five years

Most of undernourished children, 71.2% (*n* = 104), were admitted to the hospital wards from the ED-MNH, 26.7% (*n* = 39) were discharged home directly from the ED-MNH, and 2.1% (*n* = 3) died in the ED-MNH. Four undernourished children (2.9%) died in the first 24 h of hospitalization. A total of 13.3% (*n* = 18) died at the end of the 30-day follow-up (Table 3). At 24 h and at 30 days, nine (6.2%) and eleven (7.5%) patients respectively were lost to follow-up.

**Table 3:** Disposition and mortality of undernourished of children under five years

Clinical variables	Undernourished children n/N ^a^ (%)
Admitted to the ward	104/146 (71.2)
Discharge from ED	39/146 (26.7)
Died in ED	3/146 (2.1)
24-h hospital mortality	4/137 (2.9)
48-h hospital mortality	6/137 (4.4)
30-day hospital mortality	18/135 (13.3)

^a^**N** = total patients with followup at each given time period. **N** was 146 on day of enrolment and ***N*** = 137 at 24- and 48-h, and ***N*** = 135 at 30 day followup

In undernourished children, the most common diagnoses made by emergency physicians were congenital heart disease 20.7% (*n* = 28), pneumonia 11.1% (*n* = 15), and cerebral palsy 8.9% (*n* = 12). Undernutrition (of any severity) was formally diagnosed by the emergency physician in 6.7% (*n* = 9) of the undernourished children. Diagnoses of congenital heart disease, pneumonia, and/or cerebral palsy did not have a statistically significant association with mortality in our sample. A reduced level of consciousness on arrival to the ED, with a Glasgow Coma Scale of less than fifteen, and tachycardia were statistically significant risk factors for death, with mortality rates of 16.1% (*n* = 23), and 24.6% (*n* = 35), respectively. (Table 4). There was no significant association between the severity of undernutrition and mortality.

**Table 4:** Final diagnosis and risk of death of undernourished children under 5 years

Clinical variables	Overall n/N (%)	Died n/N (%)	Survived n/N (%)	RR(95% CI)	*P* value
Provider’s diagnosis					
Congenital heart diseases	28/135 (20.7%)	2/18 (11.1%)	26/117 (22.2%)	0.5 (0.1–1.7)	0.44
Pneumonia	15/135 (11.1%)	3/18 (16.7%)	12/117 (10.3%)	1.6 (0.5–4.2)	0.69
Cerebral palsy	12/135 (8.9%)	1/18 (5.6%)	11/117 (9.4%)	0.6 (0.1–2.8)	0.93
Acute malnutrition	9/135 (6.7%)	3/18 (16.7%)	6/117 (5.1%)	2.8 (1.0–6.6)	0.19
Tumours	9/135 (6.7%)	2/18 (11.1%)	7/117 (6.0%)	1.8 (0.5–5.0)	0.76
Vital signs, point of care test					
Bradypnea	8/133 (6.0%)	1/18 (5.6%)	7/115 (6.1%)	0.9 (0.2–3.9)	> 0.99
Tachycardia	34/134 (25.3%)	10/18 (55.5%)	24/116 (21.6%)	3.7 (1.6–8.3)	< 0.01
Hypotension	8/21 (38.1%)	3/8 (37.5%)	5/13 (38.5%)	1.0 (0.3–2.8)	> 0.99
Low AVPU score (GCS < 15)	21/132 (15.6%)	8/18 (44.4%)	13/114 (11.4%)	4.2 (1.9–9.1)	< 0.01
Fever (T > 37.5 °C)	28/135 (20.7%)	5/18 (27.8%)	23/117 (19.7%)	1.5 (0.6–3.5)	0.63
Hypoglycaemia (RBG < 3.5)	3/55 (5.5%)	1/16 (6.3%)	2/39 (5.1%)	1.2 (0.2–3.3)	> 0.99
Hypokalaemia	10/35 (28.6%)	3/10 (30.0%)	7/25 (28.0%)	1.1 (0.3–3.0)	0.90
Undernutrition classification					
Mild type	39/135 (28.9%)	5/18 (27.8%)	34/117 (29.1%)	1.0 (0.4–2.3)	> 0.99
Moderate type	49/135 (36.3%)	5/18 (27.8%)	44/117 (37.6%)	0.7 (0.3–1.7)	0.59
Severe type	47/135 (34.8%)	8/18 (44.4%)	39/117 (33.3%)	1.5 (0.6–3.4)	0.51

## Discussion

This study showed that a large proportion (34.1%) of acutely ill children presenting to the ED-MNH suffer from undernutrition. As has been observed in previous in-hospital patient studies, in our study most undernourished children were males under 2 years of age [1, 17, 18] and most caretakers were biological mothers with low socioeconomic status [11, 19–21]. Interestingly, we found an overwhelming number of primary caretakers had at least primary school education, which is in contrast with previous studies, as education has been shown to be protective in the development of childhood undernutrition [9, 19]. However, we did not have a control group of non-undernourished children to compare the relative rates of caretaker education. We also observed high numbers of children with congenital heart diseases and cerebral palsy as the most common chronic diseases, which have been reported to be associated with poor feeding practises leading to undernutrition [22, 23]. As MNH is a tertiary referral centre that specifically takes care of children with congenital heart disease and cerebral palsy, the high number of children with undernutrition with these chronic diseases in our study is unlikely to reflect the burden of undernourished children in most acute care settings in Tanzania.

The high frequency of symptoms of fever and cough found in our study is similar to what is reported in previous studies among undernourished children presenting to acute intake areas and EDs in sub-Saharan Africa [1, 9, 24, 25]. These symptoms could be the result of acute illness due to sepsis, pneumonia, upper respiratory infections, and malaria, all of which are believed to be associated with acute undernutrition [1, 9]. Furthermore, the findings of hypoxia, tachycardia, tachypnoea, and hypotension are similar to observations made in previous studies in Kenya, which also found such variables to be risk factors for mortality among undernourished children [19, 26, 27]. The abnormal routine investigation findings we observed, such as low blood glucose, hypokalaemia, hyponatremia, and low haemoglobin are also similar to what has been observed in previous studies of undernourished children in Kenya [19, 28, 29].

Our study found that although the true percentage of acute undernourished patients was 34.1%, emergency physicians only wrote down a diagnosis related to acute undernutrition in 6.7% of children. This low recognition rate is concerning since the care rendered to undernourished children should differ from their non-undernourished counterparts [4, 12, 30]. In order to optimise patient outcomes, providers need to give special consideration to the possibility of undernutrition with the use of a screening tool at EDs [1, 3]. We also found that undernourished children are likely to be hospitalised. This may be attributed to the fact that the majority of these children presented with fever and cough, which might signify sepsis and pneumonia, making these children less likely to be discharged from the ED.

We found a 30-day mortality of 13.3% in undernourished children in our study, similar to previous studies in LMICs, which report mortality ranges from 3.4 to 40.1% of undernourished children under five years of age [1, 10, 27, 31]. Of these, 2.9% of deaths occurred within the first 24 h of hospitalisation, a time period that is often associated with initial management in the ED. The observed overall mortality is unacceptably high compared to the WHO accepted rate of less than 5%, and this might result from late recognition, variability in treatment, and late arrival to care [1, 4, 12, 30, 32]. In our study we did find that reduced levels of consciousness (GCS < 15) and tachycardia were associated with a statistically significant increase in mortality among undernourished children, similar to observations made in the *Maitland* et al. study [18]. However, our study was not powered to evaluate for clinical features that increased the risk of mortality. An additional consideration for future analysis includes the relative hospital length of stay and resource utilization among children who are undernourished and those who are not. Finally, future studies should focus on the impact of interventions aimed at increasing identification and early treatment of undernutrition in the ED on patient disposition and outcomes.

## Limitations

In this study, the primary goal was to describe the cohort of undernourished children presenting to the ED-MNH. As we did not recruit a control group, we are unable to compare between groups. Furthermore, this study was a single centre study at an urban tertiary referral ED. Therefore, the results may not be generalizable to other hospitals. Most undernourished children in Tanzania are treated at health centres and district hospitals, so those that arrive at MNH may be more complex by the nature of the referral system.

There was low recognition of undernutrition by ED physicians in our study, and low recording of the presence or absence of oedema that would have helped up to define the severity of the undernutrition. We used what was recorded in the chart to measure recognition of undernutrition. ED physicians might have recognised undernutrition without documenting it as an ED diagnosis. Finally, while we report univariate analysis of the clinical factors associated with mortality among the undernourished children, the sample size did not allow for multivariate analysis.

## Conclusion

More than one third of acutely ill children below the age of five years who presented to the national hospital’s ED in Tanzania were undernourished and this group had a high admission and mortality rate. Although the rate of undernutrition was high, emergency medicine physicians rarely made the diagnosis of undernutrition formally, as evidenced by documentation in the chart. Emergency physicians need to consider the diagnosis of undernutrition to ensure proper patient management and disposition of these patients. Future studies should focus on the impact of interventions aimed at improving the identification of undernutrition in the ED and the effects of ED management on these children, their dispositions and outcomes.

## Data Availability

The structured questionnaire and full dataset supporting the conclusions of this article are available from the authors on request.

## Abbreviations

AVPU Scale: Alert, Voice, Pain and Unresponsive
CI: Confidence Interval
ED: Emergency Department
GCS: Glasgow Coma Scale.
LMIC: Low- and Middle-Income Countries
MNH: Muhimbili National Hospital
RR: Relative Risk
SD: Standard Deviation
USA: United States of America
WHO: World Health Organization
